# Diagnostic Accuracy of the Faecal Immunochemical Test in Triage of Symptomatic Patients Referred for Colonoscopy: A Prospective Single-Centre Study

**DOI:** 10.1155/cjgh/2883395

**Published:** 2025-08-22

**Authors:** T. Matthews, M. O'Sullivan, A. Billur, F. Janjua, A. Aftab, F. Zeb, G. Courtney

**Affiliations:** ^1^Department of Gastroenterology, St Luke's General Hospital, Kilkenny, Ireland; ^2^Department of Gastroenterology, Mater Misericordiae University Hospital, Dublin, Ireland; ^3^School of Medicine, University College Dublin, Dublin, Ireland

**Keywords:** colonoscopy triage, colorectal cancer, diagnostic accuracy, endoscopy services, faecal immunochemical test (FIT), health service evaluation, secondary care, symptomatic patients

## Abstract

**Aim:** The faecal immunochemical test (FIT) is endorsed by NICE for triaging symptomatic patients referred from primary care. This prospective diagnostic accuracy study assessed the performance of FIT in detecting significant colorectal pathology among symptomatic patients referred for colonoscopy in secondary care.

**Method:** Between May 2023 and May 2024, FIT kits were distributed to 1296 adult patients referred for lower gastrointestinal (GI) endoscopy. A FIT threshold of ≥ 50 ng/mL prompted urgent colonoscopy; values < 50 ng/mL led to outpatient assessment unless Health Service Executive Priority 1 criteria were met. A complete colonoscopy served as the reference standard.

**Results:** A total of 1113 patients (86%) returned valid FIT results; 215 (19%) were FIT positive. FIT-positive patients were significantly older than FIT-negative patients (58 vs. 54 years, *p* < 0.01). Among FIT-positive patients, 177 (82%) underwent colonoscopy, compared with 139 (15%) of FIT-negative patients. Colorectal cancer was detected in 20 FIT-positive patients and in none of the FIT-negative group, yielding a sensitivity and negative predictive value (NPV) of 100% (95% CI for sensitivity: 83–100 and NPV: 97–100). The area under the receiver operating characteristic (ROC) curve was 0.868 (95% CI: 0.82–0.91). For advanced polyps and inflammatory pathology, sensitivities were 77% and 89%, with NPVs of 98% for both. The mean time to endoscopy was shorter in FIT-positive patients (7 vs. 21 weeks, *p* < 0.01).

**Conclusion:** FIT demonstrates excellent sensitivity for colorectal cancer and may serve as a safe, effective triage tool in symptomatic patients, helping optimise endoscopy services in resource-limited settings.

**Summary**

• What does this paper add to the literature?
◦ This study demonstrates that faecal immunochemical testing safely and effectively triages symptomatic patients for colonoscopy in secondary care. By confirming high diagnostic accuracy in a real-world setting, it supports FIT's broader integration into healthcare systems to optimise endoscopy services and improve timely colorectal cancer detection.

## 1. Introduction

The Irish National Gastrointestinal Endoscopy Quality Improvement (NEQI) Programme reported a 27.4% increase in endoscopy workload between 2016 and 2019, far exceeding the 10.5% growth in the population aged over 64 years during the same period [1, 2]. Similar trends have been identified in the United Kingdom [3]. Much of this rising demand relates to the investigation of lower gastrointestinal (GI) symptoms, which are often nonspecific; notably, 97% of the patients referred for urgent colonoscopy on symptomatic grounds do not have colorectal cancer (CRC) [4].

The faecal immunochemical test (FIT) is a quantitative assay that detects intact human haemoglobin and its early degradation products in stool [5]. Elevated faecal haemoglobin concentrations are associated with CRC and other colorectal pathology [6]. FIT is noninvasive, easily completed at home, and facilitates postal submission for analysis, making it a practical tool for triage.

*BowelScreen* is Ireland's national CRC screening programme, targeting asymptomatic individuals aged 59–69 years. Eligible individuals are invited by post to complete a home-based FIT. Those with a positive result (≥ 225 ng/mL, ≥ 45 μg/g) are referred for colonoscopy through designated screening centres [7]. In contrast, the United Kingdom and Irish guidelines recommend a lower threshold of ≥ 50 ng/mL (10 μg/g) to guide referral decisions in symptomatic patients [8–11]. Within secondary care, FIT is increasingly used to stratify referrals that do not meet urgent endoscopy criteria [12]. In the Irish healthcare context, secondary care is defined as specialist hospital-based services provided following referral from family medicine-based primary care. However, real-world evidence supporting its diagnostic accuracy in this context remains limited.

This study aimed to evaluate the performance of FIT in triaging symptomatic patients referred for colonoscopy in secondary care.

## 2. Methods

### 2.1. Study Design and Setting

This was a prospective, single-centre diagnostic test accuracy study conducted between 1 May 2023 and 31 May 2024 at St Luke's General Hospital, Kilkenny. The objective was to evaluate the performance of faecal immunochemical testing as a triage tool for symptomatic patients referred for lower GI endoscopy and to evaluate its diagnostic accuracy for detecting significant colorectal pathology.

### 2.2. Participants

All consecutive adult referrals for lower GI endoscopy (*n* = 1765) were assessed for inclusion. A total of 1704 (96.5%) were referred from primary care (general practitioners) and 61 (3.5%) were referred from hospital-based consultants within secondary care. Patients were contacted by telephone to explain the pilot programme and obtain verbal consent to receive a FIT kit by post. FIT kits were posted to 1296 patients who consented to participate and were returned via the same method. Patients who declined FIT (*n* = 469), did not return kits (*n* = 112) or submitted inadequate samples (*n* = 15) were referred for an outpatient assessment unless clinical details mandated an urgent colonoscopy as per Health Service Executive (HSE) Priority 1 criteria [10]. Patients who declined FIT (*n* = 469) did not consent to receive a kit, while those who did not return kits (*n* = 112) had initially agreed but did not complete or return the test. HSE Priority 1 criteria are described in detail at reference [10] and at Table 1 but briefly include alarm features (interpreted in the context of age and family history of CRC or inflammatory bowel disease) comprising rectal bleeding, altered bowel habit, unexplained weight loss, unexplained iron deficiency anaemia, a palpable mass, abnormal abdominal imaging or suspected inflammatory bowel disease. Only patients who underwent a complete colonoscopy were included in diagnostic accuracy analysis.

### 2.3. Index Test (FIT)

FIT was performed using a quantitative immunoassay (Eurofins Biomnis; OC-Sensor Pledia, Latex Agglutination Immunoturbidimetry), with a positivity threshold set at ≥ 50 ng/mL (10 μg/g) of faecal haemoglobin. Patients with FIT ≥ 50 ng/mL were triaged for urgent colonoscopy. Those with FIT < 50 ng/mL were referred for outpatient review unless HSE Priority 1 criteria mandated direct colonoscopy [10].

### 2.4. Reference Standard (Complete Colonoscopy)

A complete colonoscopy was chosen as the reference standard due to its established role in CRC diagnosis. A complete colonoscopy was defined as a procedure where the endoscope reached the terminal ileum, caecum, neoterminal ileum or neoterminal ileocolonic anastomosis. Procedures where progression to these landmarks was prevented by a subsequently confirmed CRC were also included within this definition. Colonoscopies that did not meet these criteria (*n* = 20) were excluded from the diagnostic performance analysis.

Procedures were performed according to standard HSE protocols by experienced endoscopists, who were not blinded to FIT results. Findings were categorised as colorectal malignancy, advanced polyps (> 1 cm), inflammatory pathology (ileitis, colitis or proctitis) or no significant abnormality.

### 2.5. Outcomes

Primary outcomes included the sensitivity, specificity, positive predictive value (PPV), negative predictive value (NPV) and area under the receiver operating characteristic (ROC) curve for FIT in detecting malignancy. Secondary outcomes included FIT performance for advanced polyps, inflammation and any significant bowel pathology.

### 2.6. Sample Size Consideration

This study was designed as a pragmatic evaluation, without prospective power calculation. However, a post hoc estimate showed that 35 cancer cases would be needed to estimate sensitivity of 90% with ±10% precision at 95% confidence. Based on an expected CRC prevalence of 2%-3%, this corresponds to a cohort size of approximately 1200–1750 patients, which aligns with our study population. The 20 CRC cases observed provide reasonable precision for estimating diagnostic performance.

### 2.7. Statistical Analysis

Statistical analysis was conducted using Microsoft Excel (Version 16.16.27) and RStudio for macOS (Version 2024.12.1 + 563). Continuous variables were analysed using a Student's *t*-test (equal variance) and categorical variables via binary logistic regression. Confidence intervals for diagnostic metrics were calculated at the 95% level. ROC curves and Youden's J statistics were generated in RStudio. We used the STARD reporting guideline [13] to draft this manuscript and the STARD reporting checklist [14] when editing.

## 3. Results

### 3.1. Study Population and FIT Completion

**Figure 1:**
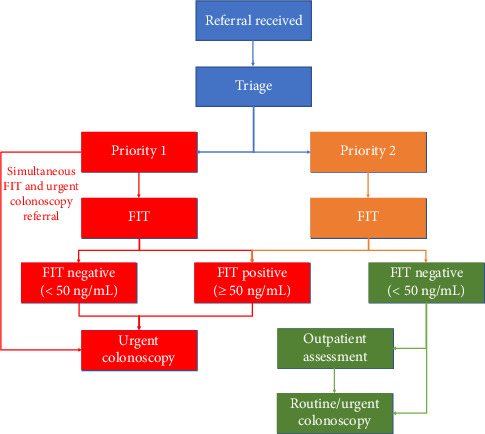
Local triage pathway for the use of FIT in colonoscopy referral prioritisation. “Priority 1” is defined according to the HSE acute operations endoscopy programme triage guidance for upper and lower gastrointestinal endoscopic procedures (Table 1), while “Priority 2” includes all referrals not meeting Priority 1 criteria [10].

**Figure 2:**
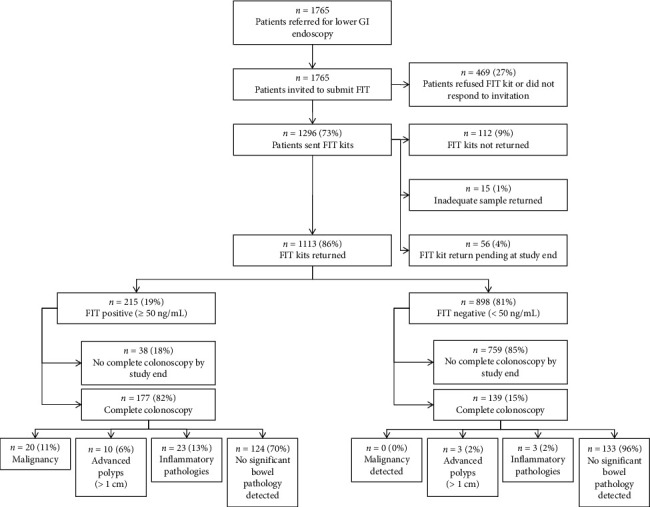
Flow of participants through the study, including referral for faecal immunochemical testing (FIT), return of test results and colonoscopy outcomes. Patients who did not return FIT or did not undergo a complete colonoscopy were excluded from diagnostic accuracy analysis.

During the 13-month study period, 1765 adult patients were referred for lower GI endoscopy. Of these, 1296 (73%) agreed to complete a FIT and were sent kits by post. A total of 1113 patients (86%) returned valid results; 112 (9%) did not return kits, 56 (4%) were pending at study end and 15 (1%) provided inadequate samples. FIT triage and participation pathways are shown in Figures 1 and 2.

### 3.2. FIT Results and Baseline Characteristics

Among patients with returned FIT results, 215 (19%) were FIT positive (≥ 50 ng/mL) and 898 (81%) were FIT negative. FIT-positive patients were significantly older than FIT-negative patients (mean age 58.4 vs. 53.5 years; *p* < 0.01), but gender was not a significant predictor of FIT positivity (48.4% vs. 41.8% male; *p*=0.08). Full demographic data are summarised in Table 2.

### 3.3. Colonoscopy and Time to Procedure

By study end, 177 (82%) FIT-positive patients and 139 (15%) FIT-negative patients had undergone a complete colonoscopy. A total of 20 patients with FIT results who had undergone incomplete colonoscopy were excluded from the analysis. No malignancies were detected during these incomplete procedures. At study close, none of these 20 patients had undergone a repeat procedure.

Among FIT-negative patients, those selected for colonoscopy were chosen based on clinician judgement at outpatient review or were sent directly to colonoscopy if they met HSE Priority 1 criteria [10]. FIT-positive patients had significantly shorter wait times for colonoscopy (mean 6.5 weeks vs. 21.2 weeks; *p* < 0.01). No clinically significant adverse events were reported.

### 3.4. Diagnostic Performance for Malignancy

CRC was detected in 20 of 177 FIT-positive patients who underwent complete colonoscopy (11.3%) and in none of the 139 FIT-negative patients.

This yielded a sensitivity of 100% (95% CI: 83.2–100), specificity of 47% (95% CI: 41.2–52.8), and NPV of 100% (95% CI: 97.4–100) (Table 3). The number needed to scope (NNS) in the FIT-positive cohort to detect one cancer was 8.85.

The mean FIT level in patients with malignancy was significantly higher than in those without (816 ng/mL vs. 250 ng/mL; mean difference 566 ng/mL, 95% CI: 398–733; *p* < 0.01).

**Figure 3:**
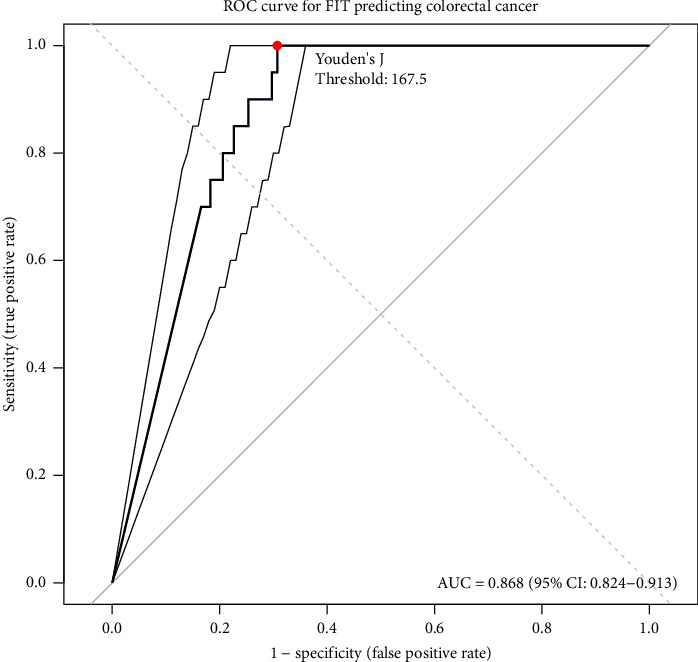
Receiver operating characteristic (ROC) curve for FIT at a threshold of ≥ 50 ng/mL as a diagnostic tool for colorectal malignancy. Area under the curve (AUC): 0.868 (95% CI: 0.824–0.913). Maximum test performance (sum of sensitivity and specificity) was achieved at a threshold of 167.5 ng/mL in this cohort.

ROC analysis yielded an AUC of 0.868 (95% CI: 0.824–0.913) (Figure 3). Youden's J statistic identified an optimal FIT threshold of 167.5 ng/mL, maximising sensitivity to 100% and improving specificity to 69.3%.

Although only 15% of FIT-negative patients underwent colonoscopy, this subset likely represented a higher-risk group selected by clinical judgement. No malignancies were identified among them.

### 3.5. Diagnostic Performance for Advanced Polyps

After excluding malignancy, advanced polyps (> 1 cm) were detected in 10 FIT-positive patients (6.4%) and 3 FIT-negative patients (2.2%). One FIT-positive patient had a polyp > 3 cm; none were detected in FIT-negative patients.

FIT sensitivity for detecting advanced polyps was 77% (95% CI: 46.2–95), specificity 48.1% and NPV 97.8% (Table 3).

### 3.6. Diagnostic Performance for Inflammatory Pathology

Inflammatory pathology (ileitis, colitis or proctitis) was found in 23 FIT-positive patients (13.0%) and 3 FIT-negative patients (2.2%). This yielded a sensitivity of 88.5%, specificity of 46.9% and NPV of 97.8% for FIT in detecting inflammation (Table 3).

### 3.7. Diagnostic Performance for Any Significant Bowel Pathology

Any significant bowel pathology (malignancy, advanced polyp or inflammation) was found in 53 FIT-positive patients (30.0%) and 6 FIT-negative patients (4.3%). This yielded a sensitivity of 89.8%, specificity of 51.8% and NPV of 95.7% for FIT in detecting any significant bowel pathology (Table 3).

## 4. Discussion and Conclusions

### 4.1. Principal Findings

This prospective, single-centre study demonstrates that faecal immunochemical testing at a threshold of ≥ 50 ng/mL offers excellent diagnostic performance in triaging symptomatic patients referred for lower GI endoscopy. FIT achieved a sensitivity and NPV of 100% for CRC within our cohort, supporting its role in identifying patients most likely to benefit from urgent colonoscopy.

FIT also performed well for detecting advanced polyps, inflammatory pathology and any significant bowel pathology, with NPVs of 97.8%, 97.8% and 95.7%, respectively, highlighting its broader utility in excluding significant colorectal disease. In addition, FIT-positive patients underwent colonoscopy significantly sooner than FIT-negative patients, suggesting improved efficiency in care pathways.

### 4.2. Comparison With Existing Literature

Our findings align closely with larger studies. The multicentre National Institute for Health and Care Excellence (NICE) FIT study reported a CRC prevalence of 3.3%, a malignancy NPV of 99.6% and an ROC AUC of 0.93 [15]. A meta-analysis of over 25,000 patients similarly estimated pooled sensitivity and specificity of 88.7% and 80.5%, respectively, for FIT at the same threshold [16]. Our AUC of 0.868 supports the test's strong discriminatory capacity in a real-world secondary care setting.

Colonoscopy remains the reference standard for CRC diagnosis but has a reported sensitivity of 94.7% [17]. FIT, while not a replacement, provides a noninvasive and scalable adjunct to risk stratify patients in resource-constrained services.

### 4.3. Strengths and Limitations

Strengths of this study include its prospective design, high FIT uptake (86%) and real-world clinical setting. The study addresses a critical operational issue: how to prioritise endoscopy resources without compromising diagnostic safety.

However, there are limitations. Only 15% of FIT-negative patients underwent colonoscopy during the study period, which may introduce spectrum bias. These patients were selected based on clinical suspicion and thus likely represent a higher-risk subgroup. Reassuringly, no malignancies were detected among them. The study's single-centre nature may limit generalisability. At the time of study closure, no CRCs had been diagnosed among FIT-negative patients who did not undergo colonoscopy. However, extended follow-up will be essential to confirm the long-term safety of FIT-based triage.

### 4.4. Other Clinical Implications

Test efficiency analysis suggested an optimal FIT threshold of 167.5 ng/mL for balancing sensitivity and specificity. While this may reduce colonoscopy demand, it risks reduced sensitivity. Given the high stakes of missed cancers, maintaining the threshold at ≥ 50 ng/mL remains appropriate, particularly in symptomatic populations.

## 5. Conclusion

While colonoscopy remains the reference standard for CRC diagnosis, its sensitivity is not absolute [17], and access is often limited by service capacity. In this context, the faecal immunochemical test provides a reliable, noninvasive and cost-effective triage tool that closely approximates the diagnostic performance of colonoscopy for CRC. Its implementation can enhance the prioritisation of high-risk patients, reduce unnecessary procedures and alleviate pressure on overstretched endoscopy services.

Broader adoption of FIT in symptomatic referral pathways may also enable capacity gains for public health initiatives, including potential expansion of asymptomatic CRC screening programmes. Ultimately, integrating FIT into clinical workflows represents a pragmatic, evidence-based approach to improving diagnostic efficiency and outcomes in an ageing population with growing demand for CRC diagnostics.

## Figures and Tables

**Table 1:** Health Service Executive Priority 1 urgent colonoscopy criteria.

Age ≥ 60 years	Age ≥ 40 years	Age < 40 years	Any age
Rectal bleeding > 6 weeks OR Change in bowel habit > 6 weeks OR Unexplained significant weight loss with symptoms suggestive of colorectal cancer	Rectal bleeding AND Change in bowel habit for > 6 weeks	Unexplained rectal bleeding AND/OR change in bowel habit AND A family history of colorectal^∗^ OR Inflammatory bowel disease	Palpable abdominal or rectal mass Unexplained iron deficiency anaemia^∗∗^ Significant weight loss with symptoms suggestive of underlying colorectal cancer Abnormal abdominal imaging Suspected inflammatory bowel disease^∗∗∗^

^∗^One first-degree relative diagnosed with colorectal cancer under the age of 50; two or more relatives with colorectal or endometrial cancer, one of these should be a first-degree relative of the patient, and they should be first-degree relatives of each other; family history of colorectal cancer syndrome such as Lynch syndrome or polyposis.

^∗∗^Male (any age) ≤ 11 g/100 mL and female (nonmenstruating) ≤ 10 g/100 mL, concordant serum ferritin.

^∗∗∗^Patients with symptoms suggestive of new onset inflammatory bowel disease should receive urgent investigation. Application of noninvasive testing such as faecal calprotectin and imaging studies will assist individuals. Patients aged < 40 years with persistent bloody diarrhoea should be referred for urgent (P1) sigmoidoscopy. Patients aged < 40 years with isolated rectal bleeding should be referred for routine (P2) sigmoidoscopy.

**Table 2:** Patient characteristics.

Population characteristics (all patients with FIT results)	FIT positive (*n* = 215, 19%)	FIT negative (*n* = 898, 81%)	*p* value
Male: *N* (%)	104 (48.4)	375 (41.8)	*p*=0.08
Female: *N* (%)	111 (51.6)	523 (58.2)	
Age: mean, median (IQR)	58.4, 59 (47.5–72)	53.5, 54 (42–65)	*p* < 0.01
FIT result (ng/mL): mean, median (IQR)	480.9, 275 (118.5–1000)	4.4, 0 (0–4)	*p* < 0.01

**Population characteristics (colonoscopy completed)**	**FIT positive (*n* = 177, 82%)**	**FIT negative (*n* = 139, 15%)**	**p** **value**

Male: *N* (%)	86 (48.6)	68 (48.9)	*p*=0.95
Female: *N* (%)	91 (51.4)	71 (51.1)	
Age: mean, median (IQR)	57.8, 59 (45–71)	57.4, 60 (48–66)	*p*=0.8
FIT result (ng/mL): mean, median (IQR)	505.8, 328 (119–1000)	6.2, 0 (0–5)	*p* < 0.01
Weeks to endoscopy from receipt of referral: mean, median (IQR)	6.5, 5 (4–6.4)	21.2, 18 (9.4–32.4)	*p* < 0.01

**Population characteristics (malignancy)**	**Malignancy (*n* = 20, 6%)**	**No malignancy (*n* = 296, 94%)**	**p** **value**

Male: *N* (%)	11 (55)	143 (48.3)	*p*=0.56
Female: *N* (%)	9 (45)	153 (51.7)	
Age: mean, median (IQR)	68.1, 71.5 (63.25–76.5)	56.9, 59 (47–68)	*p* < 0.01
FIT result (ng/mL): mean, median (IQR)	816.1, 1000 (705.5–1000)	250.2, 54 (0–297.25)	*p* < 0.01
Weeks to endoscopy from receipt of referral: mean, median (IQR)	8.7, 5.7 (4–8.5)	13.3, 6.9 (4.7–18.8)	*p*=0.05

*Note:* FIT positivity threshold was set at 50 ng/mL.

**Table 3:** Diagnostic performance metrics for FIT at a threshold of ≥ 50 ng/mL.

	Sensitivity (%)	Specificity (%)	Positive predictive value (%)	Negative predictive value (%)
Malignancy	100 (83.2–100)	47 (41.2–52.8)	11.3 (10.3–12.4)	100 (97.4–100)
Advanced polyp (> 1 cm)	77 (46.2–95)	48.1 (42.1–54.1)	6.4 (4.7–8.6)	97.8 (94.3–99.2)
Inflammation	88.5 (69.9–97.6)	46.9 (41–52.8)	13 (11.1–15.1)	97.8 (94–99.3)
Any significant bowel pathology	89.8 (79.2–96.2)	51.8 (45.5–58)	29.9 (26.8–33.3)	95.7 (91.1–98)

	**Malignancy (no.)**	**No malignancy (no.)**	**Total (no.)**	

FIT positive	20	157	177	
FIT negative	0	139	139	
Total	20	296	316	

	**Advanced polyp (> 1 cm) (no.)**	**No advanced polyp (> 1 cm) (no.)**	**Total (no.)**	

FIT positive	10	147	157	
FIT negative	3	136	139	
Total	13	283	296	

	**Inflammation (no.)**	**No inflammation (no.)**	**Total (no.)**	

FIT positive	23	154	177	
FIT negative	3	136	139	
Total	26	290	316	

	**Any significant bowel pathology (no.)**	**No significant bowel pathology (no.)**	**Total (no.)**	

FIT positive	53	124	177	
FIT negative	6	133	139	
Total	59	257	316	

*Note:* Values in parentheses indicate 95% confidence intervals.

## Data Availability

The data that support the findings of this study are available from the corresponding author upon reasonable request. No identifiable patient data are reported.
